# Thiomolybdate Clusters: From Homogeneous Catalysis to Heterogenization and Active Sites

**DOI:** 10.1002/adma.202305730

**Published:** 2023-12-02

**Authors:** Samar Batool, Marcel Langer, Stephen Nagaraju Myakala, Magdalena Heiland, Dominik Eder, Carsten Streb, Alexey Cherevan

**Affiliations:** ^1^ Institute of Materials Chemistry TU Wien Getreidemarkt 9/BC/02 Vienna 1060 Austria; ^2^ Institute of Inorganic Chemistry I Ulm University Albert‐Einstein‐Allee 11 89081 Ulm Germany; ^3^ Department of Chemistry Johannes Gutenberg University Mainz Duesbergweg 10‐14 55128 Mainz Germany

**Keywords:** electrocatalysis, heterogenization, hydrogen evolution, molybdenum sulfide, photocatalysis, thiomolybdate

## Abstract

Thiomolybdates are molecular molybdenum‐sulfide clusters formed from Mo centers and sulfur‐based ligands. For decades, they have attracted the interest of synthetic chemists due to their unique structures and their relevance in biological systems, e.g., as reactive sites in enzymes. More recently, thiomolybdates are explored from the catalytic point of view and applied as homogeneous and molecular mimics of heterogeneous molybdenum sulfide catalysts. This review summarizes prominent examples of thiomolybdate‐based electro‐ and photocatalysis and provides a comprehensive analysis of their reactivities under homogeneous and heterogenized conditions. Active sites of thiomolybdates relevant for the hydrogen evolution reaction are examined, aiming to shed light on the link between cluster structure and performance. The shift from solution‐phase to surface‐supported thiomolybdates is discussed with a focus on applications in electrocatalysis and photocatalysis. The outlook highlights current trends and emerging areas of thiomolybdate research, ending with a summary of challenges and key takeaway messages based on the state‐of‐the‐art research.

## Graphical abstract

This review highlights recent developments in the use of molecular molybdenum sulfide clusters, thiomolybdates as reactive sites in photo‐ and electrochemical catalysis. Mechanistic details and comparison of reactivity and stability are reported with a focus on the hydrogen evolution reaction.

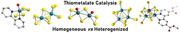

## 1 Introduction

Extraction, processing, and combustion of fossil fuels are the cornerstones of our modern global economy. However, fossil feedstocks are finite, and their use is the main driver of greenhouse gas emissions and climate change. The continuously increasing energy demand poses massive challenges for the global economy and underlines the need to transition from fossil to renewable energy systems. One promising alternative fuel is hydrogen (H_2_) as it has a high gravimetric energy density and can be produced from water by chemical, photochemical, and electrochemical means. However, traditional methods of H_2_ production, such as steam reforming, need to be replaced with sustainable technologies including water electrolysis or photocatalysis, which enable the direct production of “green”, sustainable H_2_ using renewable energy. Thus, the development of efficient and robust catalytic systems for the splitting of water is one of the grand current challenges in chemistry. Cooperative and interdisciplinary research efforts are required to design earth‐abundant and high‐performance electro‐ and photocatalysts able to perform the hydrogen evolution reaction (HER) with high efficiency, selectivity, and stability.

**Scheme 1:**
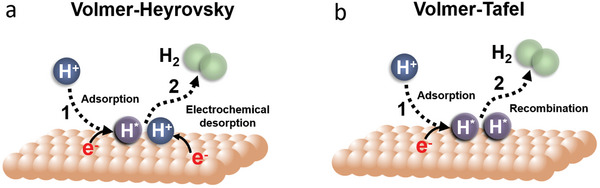
Simplified HER pathways taking place under acidic conditions. a) Volmer–Heyrovsky mechanism in which H_2_ formation proceeds via PCET and electrochemical desorption steps, b) Volmer–Tafel mechanism in which two surface‐adsorbed hydrogen atoms (H*) recombine to form H_2_.

HER involves the proton‐coupled transfer of two electrons and occurs in two steps. The first step of HER is the Volmer reaction, which involves electron transfer to adsorbed H^+^ ions to form hydrogen intermediates (H*) bound to the catalyst. After this, the formation of molecular H_2_ can occur via two reaction pathways, depending on the surface coverage of H*. If the surface coverage is low, the H_2_ formation will proceed by electrochemical hydrogen desorption—termed as Heyrovsky reaction—where a proton‐coupled electron transfer (PCET) results in the formation and release of H_2_ (**Scheme** **1**a). In contrast, if the surface coverage of H* is high, this second step proceeds via the Tafel reaction, where two neighboring surface‐bound hydrogen atoms H* are coupled to give dihydrogen (**Scheme** 1b). Independent of the reaction mechanism, it is evident that H* is involved in all reaction steps, hence the Gibbs free energy of hydrogen adsorption (Δ*G*_H*_) becomes an important indicator for an efficient HER catalyst. As postulated in the Sabatier principle,^[^ ^1^ ^]^ and practically demonstrated by Nørskov et al.^[^ ^2^, ^3^ ^]^ the bonding strength between the catalyst surface and hydrogen atoms should neither be too weak nor too strong, implying that optimal bonding is a key to achieving optimized HER rates.

Many compounds have demonstrated HER activity, including noble metals, metal oxides, nitrides, and sulfides.^[^ ^4^ ^]^ In this respect, molybdenum sulfides (MoS_2_) have emerged as promising earth‐abundant alternatives to the state‐of‐the‐art Pt HER catalysts. MoS_2_‐based catalysts are long known as a class of industrial catalysts widely used in the world for petroleum refining processes such as hydrodesulfurization (HDS), hydrodeoxygenation (HDO), and hydrodemetallization (HDM) reactions.^[^ ^5^ ^]^ Importantly, these processes share the dihydrogen activation steps required for the following hydrogenation or hydrogenolysis reactions, which highlights the H_2_ activation reactivity of MoS_2_ with relevance for HER. Despite these widespread commercial applications of MoS_2_‐based catalysts, early experiments using bulk crystalline MoS_2_ revealed poor HER performance.^[^ ^6^ ^]^ However, later studies showed that nanostructuring of MoS_2_ results in HER activity levels approaching those of Pt.^[^ ^7^ ^]^ Also, amorphous molybdenum sulfides (a‐MoS_x_) as well as nonstoichiometric molybdenum sulfides (MoS_2±__x_) have been shown to exhibit suitable hydrogen adsorption centers and promising HER performance.^[^ ^8^, ^9^ ^]^ The demonstration that edge‐sites of MoS_2_ sheets are likely HER active sites^[^ ^10^ ^]^ triggered further interest in thiomolybdate clusters, which can be seen as molecular analogs of MoS_2_. Their well‐defined molecular structure and composition make it possible to probe their active centers and study their (de)activation pathways, ultimately delivering atomistic insights on the performance of a variety of MoS_2_‐based catalysts. This is nicely exemplified by early developments in inorganic molecular thiochemistry, which were stimulated by the needs of the petrochemical industries. In this context, molecular transition metal sulfides have been studied to gain insights into reactions that occur on the surfaces of heterogeneous catalysts. These studies involve the investigation of model feedstock molecules and the activation of dihydrogen with transition metal sulfur sites, shedding light on potential modes of binding for H_2_ and thiophenes.^[^ ^11^ ^]^

Research progress in thiomolybdate HER activity until 2018 has been summarized by Streb and colleagues in their recent review.^[^ ^12^ ^]^ The authors pointed out that thiomolybdates can act as models for two proposed active site mechanisms. The “sulfide/disulfide” mechanism proceeds via a Volmer–Heyrovsky process based on protonated sulfide/disulfide ligands. The “molybdenum hydride” mechanism proceeds via the formation of a Mo^V^─H moiety, so that Mo‐centered redox processes are involved in the hydrogen evolution.^[^ ^13^ ^]^ It was pointed out that careful design of thiomolybdate complexes can provide crucial information on the active sites and limitations of MoS‐based HER catalysis.

Previous reviews and book chapters have already provided a systematic look into the structure and synthesis of thiomolybdates and related inorganic compounds,^[^ ^14^, ^15^, ^16^ ^]^ described methodologies that have been used to construct oxothiometalate‐based materials^[^ ^17^, ^18^ ^]^ and reviewed early application of transition‐metal complexes with sulfide ligand and thiomolybdate clusters in catalysis^[^ ^19^ ^]^ and petrochemical industry.^[^ ^5^ ^]^ This progress report will put a particular focus on the latest developments in thiomolybdate‐based electro‐ and photocatalysts, especially with regard to combined experimental and theoretical studies to shed light on reaction mechanisms, active sites, and possible degradation and repair paths, as well as emerging strategies for heterogenization on functional substrates. Section 2 will introduce thiomolybdates from the perspective of their origin, structural variety and highlight some of the recent developments. Section 3.1 will provide a comprehensive look into the catalytic properties of thiomolybdates under homogeneous conditions in solution. Section 3.2 will examine state‐of‐the‐art research related to the identification of active sites of molecularly dissolved and surface‐supported thiomolybdate clusters. Section 4.1 will explore a variety of thiomolybdate compounds, including [Mo_2_S_12_]^2−^, [Mo_3_S_13_]^2−^ **_,_** [Mo_3_S_4_]^4+^, their analogs and derivatives, as HER electrocatalysts and will provide insights into the activity comparisons of the clusters compared to other MoS_x_‐based nanostructures. Section 4.2 will document the successful implementation of basic thiomolybdate clusters as HER co‐catalysts by combining them with a range of oxide, nitride, sulfide, and microporous supports exhibiting inorganic, organic as well as hybrid nature. Section 5 will provide a short summary of our findings and present our broad outlook aiming to provide directions for the future research.

### 1.1 Thiometalates: Structure and Recent Developments

**Figure 1:**
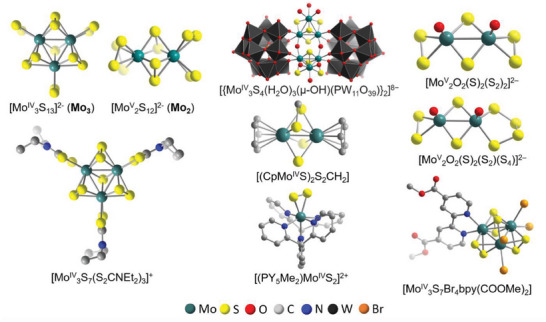
Structure illustrations of the thiomolybdate compounds described in this Review.

Pioneering work in the field of (poly)thiometalates—all‐inorganic molecular metal sulfide clusters formed by several metal centers and sulfur‐containing ligands—was in part inspired by bioinorganic studies of the enzymes nitrogenase and hydrogenase, where metal sulfide clusters were identified as active sites.^[^ ^20^, ^21^ ^]^ This insight has triggered major research into the design of artificial, biomimetic analogs.^[^ ^22^, ^23^, ^24^, ^25^, ^26^ ^]^ In ground‐breaking studies in the late 1970s, Müller and co‐workers reported the structure and characterization of the two prototype anions [Mo^V^_2_S_12_]^2−^ (**Mo_2_**)^[^ ^27^ ^]^ and [Mo^IV^_3_S_13_]^2−^ (**Mo_3_**)^[^ ^28^ ^]^ as the respective ammonium salts. Both clusters are formed by reduction of Mo^VI^ precursors (originally, ammonium heptamolybdate, (NH_4_)_6_[Mo_7_O_24_]) in (poly)sulfide‐containing aqueous solutions resulting in the isolation of crystalline products. As shown in **Figure** **1** (left), Mo^V/IV^ centers in both thiomolybdate anions are coordinated by terminal and bridging disulfide (S_2_^2−^) ligands, however, the **Mo_3_** cluster also contains an apical μ_3_‐S^2−^ ligand, which has been shown to allow initial **Mo_3_** dimerization and stacking on the way to its thermal transformation to the hexagonal MoS_2_ lattice.^[^ ^29^ ^]^ Several extensive reviews published soon after this initial work provide a comprehensive overview of the synthesis, structural archetypes, electronic configurations, and spectroscopic characteristics of these and similar anionic clusters, in particular with a focus on their relevance as bioinspired models for the active sites of nitrogenase and hydrogenase enzymes.^[^ ^30^, ^31^ ^]^

Although **Mo_2_** and **Mo_3_** can be seen as major representatives of prototypical all‐inorganic thiomolybdates, several other groups of Mo‐S‐related molecular clusters and compounds are of high relevance to this review. As such, thiomolybdates can be modified to introduce O‐containing ligands, leading to a larger group of oxothiometalate clusters (Figure 1, right).^[^ ^32^ ^]^ On the other hand, stable thiometalates with structurally similar tungsten cores, such as [W_2_O_2_S_8_]^2‐^ or [W_3_S_9_]^2−^,^[^ ^32^, ^33^ ^]^ can be studied to gain deeper understanding of the roles of sulfide/disulfide ligands and metal centers on structural and catalytic aspects of such clusters. In recent years, studies in thiometalate chemistry have moved from individual molecules to extended structures, so that 1D chains,^[^ ^34^ ^]^ metal–organic frameworks (MOFs),^[^ ^35^ ^]^ and coordination polymers^[^ ^36^ ^]^ with exciting reactivity have been reported. This opens new avenues to the design and implementation of more complex thiomolybdate (nano)structures.

Although thiomolybdates and their derivatives have been long‐known in the literature, the main interest was initially focused on their synthesis, structure, principal chemistry, and bioinorganic relevance. The in‐depth study of their hydrogen evolution activity was only triggered in 2008, when Chorkendorff and colleagues reported the electrochemical HER activity of the cubane‐type [Mo_3_S_4_(H_2_O)_9_]^4+^.^[^ ^37^, ^38^ ^]^ These groundbreaking studies have now led to a plethora of research activities, both in homogeneous and heterogeneous catalysis.

## 2 Catalytic Performance and Active Sites

In this section, we will explore the catalytic performance of prototype thiomolybdates and their derivatives under homogeneous conditions and when deposited as molecular species on heterogeneous supports and discuss proposed active sites of these species. Molecular thiomolybdate systems are ideally suited to deploy a wide range of modern analytical methods, including in situ and operando spectroscopies, to gain atomic‐level understanding of active species, reaction mechanisms, and degradation pathways. Over the last decade, pioneering mechanistic studies have utilized thiomolybdates as molecular models able to shed light on the underlying processes which govern reactivity and stability of these and more complex molybdenum sulfide‐based nanostructures. Most of these studies were focused on electrochemical or light‐driven catalysis by the prototype **Mo_3_** cluster (Figure 1, left). In the following, we will explore the principal HER reactivity of thiomolybdates in Section 3.1, while active site insights will be discussed in Section 3.2.

### 2.1 Catalytic Properties

In 2018, the groups of Min^[^ ^39^ ^]^ and Streb^[^ ^40^ ^]^ independently reported the first studies into the light‐driven HER by **Mo_3_** under homogeneous conditions. Both groups observed high catalytic activity when combining **Mo_3_** with [Ru(bpy)_3_]^2+^ as a photosensitizer (PS) and provided initial mechanistic understanding of the catalyst deactivation. Theoretical modeling of the HER process was used to rationalize the experimental data.

**Figure 2:**
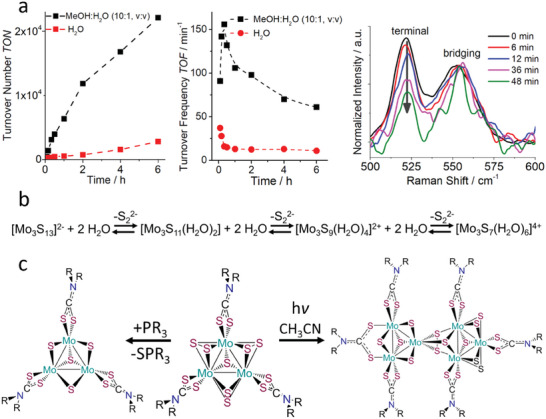
Catalytic performance under homogeneous conditions. a) Effect of solvent mixtures on light‐driven HER: turnover numbers (TONs, left) and turnover frequencies (TOFs, center) during HER catalysis indicate faster deactivation of Mo_3_ in aqueous conditions and suggest exchange of ligands followed by in situ generation of a catalytically more active species, also confirmed with in situ Raman spectroscopy (right). b) Schematic of disulfide ligand exchange with water ligands. c) Conversion of {Mo_3_S_7_}‐core to {Mo_3_S_4_}‐core containing clusters. R: methyl, ethyl, i‐butyl, benzyl. (a,b) Reproduced with permission.^[^ ^40^ ^]^ Copyright 2018, Royal Society of Chemistry. (c) Adapted with permission.^[^ ^44^ ^]^ Copyright 2019, American Chemical Society.

Streb and co‐workers focused on understanding the structural changes and deactivation pathways of **Mo_3_**. The group used Raman spectroscopy to propose the exchange of the terminal disulfide ligands of **Mo_3_** by water ligands when operating in methanol/water (MeOH/H_2_O) mixtures (**Figure** **2**a,b). Catalytic analyses and theoretical calculations indicated that the resulting **Mo_3_**‐derivatives have different HER activity, depending on the number of water ligands. This insight was used to optimize the water content of the reaction mixture, so that the most active species were stabilized, leading to turnover numbers (TONs) > 20000. The group also observed decreasing TONs with increasing **Mo_3_** concentration, which was assigned to ion pairing and colloid formation by aggregation of the anionic **Mo_3_** with the cationic PS [Ru(bpy)_3_]^2+^. In a follow‐up study using the **Mo_3_**/[Ru(bpy)_3_]^2+^/ascorbic acid solutions, the authors reported an unusual effect of ammonium ions (NH_4_^+^) in enhancing the overall HER performance of the photosystem.^[^ ^41^ ^]^ The authors performed a range of mechanistic studies and suggested that NH_4_^+^ is capable of increasing the lifetime of the photosensitizer excited state. Other contributions, including hydrogen bonding or proton management during HER, were also discussed, and the effect was demonstrated to be more general and relevant to other PS, HER‐catalysts, solvents, and sacrificial electron donors.

At the same time, Min and co‐workers analyzed the effects of varying reaction parameters on the catalytic activity of **Mo_3_**, including catalyst and photosensitizer concentration and their molar ratio. The authors also screened commonly used sacrificial electron donors such as organic amines or organic carboxylic acids. The highest light‐driven HER activity was observed for ascorbic acid, which the authors explained by its dual function as proton and electron donor. Also, the authors reported that exchange of the original PS [Ru(bpy)_3_]^2+^ with the organic dye Eosin Y resulted in a complete loss of HER activity, possibly due to weak excited‐state interactions of the PS and ascorbic acid, so that no electron transfer between the species occurs. Emission quenching studies demonstrated that ascorbic acid acts as a reductive quencher, while **Mo_3_** acts as an oxidative quencher for the excited state of [Ru(bpy)_3_]^2+^. In a subsequent study, Min and co‐workers combined **Mo_3_** with the polyoxometalate (POM) [H_4_SiW_12_O_40_] as a UV‐active photosensitizer.^[^ ^42^ ^]^ In the presence of ethanol as a sacrificial agent, and under irradiation with UV light, the POM forms a two‐electron‐reduced species capable of electron transfer to **Mo_3_**. Sustained hydrogen evolution over periods of 40 h with maximum TONs of≈6900 was reported for this molecular photosystem.

[Mo_2_S_12_]^2−^ (**Mo_2_**) is a smaller pure thiomolybdate cluster structurally closely related to **Mo_3_** (Figure 1, left). Streb and co‐workers explored the light‐driven homogeneous HER of this complex^[^ ^43^ ^]^ when combined with a PS and a sacrificial electron/proton donor. The group observed that catalyst reactivity is highly dependent on the solvent mixture used. Lower HER activity compared with **Mo_3_** was reported, with TONs ≈ 1600. Notably, **Mo_2_** shows the highest activity in pure methanol, and HER activity decreases with increasing water content of the solvent. This was rationalized by emission quenching studies which showed that increasing solvent water content leads to a less effective quenching of the [Ru(bpy)_3_]^2+^ PS by **Mo_2_**. The authors did not observe the exchange of the terminal disulfide ligands of **Mo_2_** under reaction conditions, which had been described as a major deactivation path for the reference **Mo_3_** cluster. Analysis of the main reasons for the loss of catalytic activity (studied after ca. 6 h of irradiation) showed significant PS degradation, so the addition of a second PS aliquot after the initial irradiation period could be used to re‐establish HER activity. These early results emphasize the need for replacing molecular photosensitizers with more redox‐stable absorbers—a strategy that will be discussed in Section 3.3.

In addition to studies using pure thiomolybdates such as **Mo_2_** and **Mo_3_**, other thiomolybdate derivatives have been explored under homogeneous conditions. Donahue, Schmehl and colleagues investigated the light‐driven HER activity for dithiocarbamate‐functionalized **Mo_3_** (Figure 1, left).^[^ ^44^ ^]^ In these systems, the {Mo_3_S_4_}‐core of native **Mo_3_** is retained, while the terminal disulfides are substituted by dithiocarbamate ligands, e.g., diethyl dithiocarbamate, NEt_2_CS_2_^−^. The authors used MALDI mass spectrometry to gain understanding of structural changes during catalysis. Within a few minutes of irradiation, the original species [Mo_3_S_7_(S_2_CNEt_2_)_3_]^+^ (Figure 1, left) disappears and a new species, [Mo_3_S_4_(S_2_CNEt_2_)_3_]^+^ arises. The authors propose that this new species is formed from the original cluster by exchange of the bridging disulfide ligands (S_2_^2−^) with sulfide ligands (S^2−^), so that under catalytic conditions, [Mo_3_S_7_(S_2_CNEt_2_)_3_]^+^ acts as precatalyst, forming [Mo_3_S_4_(S_2_CNEt_2_)_3_]^+^ as the initial HER‐active species. During catalysis, further speciation is observed, including the formation of dimeric species where a [Mo_3_S_7_(S_2_CNEt_2_)_3_]^+^ and a [Mo_3_S_4_(S_2_CNEt_2_)_3_]^+^ moiety are linked by a bridging sulfide ligand (Figure 2c). This observation is further evidence of the highly dynamic behavior and coordination chemistry of organo‐functionalized thiomolybdates, which has been previously discussed for the parent **Mo_3_** (vide supra).^[^ ^40^ ^]^

In a related study, Cadot and co‐workers used the {Mo_3_S_4_}‐core as an HER‐active site for homogeneous, light‐driven hydrogen evolution.^[^ ^45^ ^]^ The authors stabilized two [Mo_3_S_4_(H_2_O)_3_(µ‐OH)]^3+^ species with two [PW_11_O_39_]^8−^ polyoxometalate clusters (Figure 1, middle), and reported the light‐driven homogeneous HER of this compound using an Ir‐based PS and triethanolamine as sacrificial electron donor. The authors showed that the covalent–coordinate linkage between thiomolybdate and polyoxometalate is required for high HER activity, while physical mixtures of both components only result in low hydrogen evolution. Mechanistic studies based on steady‐state and time‐resolved optical absorption and emission spectroscopies suggested that the Ir‐based PS is reductively quenched by the electron donor and can subsequently transfer electrons to the catalyst at a high rate. Notably, high catalyst concentrations led to decreasing HER activity. The authors propose that the **Mo_3_**‐derived catalyst can absorb significant amounts of the incident photons, leading to overall suppressed rates of light‐driven HER.

These studies document the intrinsic reactivity of a variety of thiomolybdate species towards light‐driven HER under strictly homogeneous conditions and provide initial molecular‐level insights about their activity and stability. The following chapter will examine mechanistic insights on the type of active sites in thiomolybdate HER catalysts.

### 2.2 Identification of Active Sites in Thiomolybdates

Mechanistic studies on amorphous materials are notoriously challenging due to a lack of information on the material structure at the atomic level, as well as the possible presence of multiple reactive sites. However, identifying the active site and rationalizing the resulting HER mechanism are critical for advancing catalyst development, as the nature of the active site is a key factor that determines the energetics, kinetics, and stability of a catalyst.

The study of thiomolybdate clusters as molecular models for a‐MoS_x_ catalysts was fueled by a pioneering study by Tran, Artero and colleagues, who proposed that a‐MoS_x_ with high HER activity is composed of polymeric chains of **Mo_3_**.^[^ ^46^ ^]^ This report has led to massive interest in exploring thiomolybdate reactivity and rationalizing HER mechanisms along with the nature of the active sites in molybdenum sulfide hydrogen evolving catalysts. However, even for molecularly well‐defined systems such as thiomolybdates, identification of catalytically active sites is not straightforward, as active sites can depend on the reaction conditions applied (e.g., photochemical versus electrochemical processes, type of solvent, pH value, etc.).

In addition, it has been reported that thiomolybdates show highly dynamic behavior and undergo ligand exchange under typical catalytic conditions, so that different species can be present and the catalyst composition can change as a function of time (vide infra). As a result, different reactivity mechanisms are currently described in the literature based on experimental methods (e.g., spectroscopic investigations), theoretical calculations (e.g., DFT studies) and combinations of both. In the following section, we will discuss recent findings and summarize the current understanding of HER mechanisms and active sites in thiomolybdates. In addition, the summary presented in **Table** **1** aims at correlating catalyst type, experimental condition and proposed active site.

**Table 1:** Active sites of Mo‐sulfides: summary of conditions used and HER mechanisms proposed

Proposed active sites	Catalyst	Experimental conditions	Year	Ref.
Terminal disulfides				
terminal S_2_^2−^ (based on experimental data), possible role of Mo‐H mechanism (based on theory)	**Mo_3_**	Homogeneous, photochemical	2018	[40]
Terminal S_2_^2−^	**Mo_3_** and protonated species ([HMo_3_S_13_]^−^, [H_3_Mo_3_S_13_]^+^)	Homogeneous, gas‐phase CID and FT‐ICR MS^[^ ^47^ ^]^/IRMPD^[^ ^49^ ^]^	2018, 2020	[47, 49]
Bridging (di‐)sulfides				
Bridging S_2_^2−^	**Mo_3_**	Heterogeneous, electrochemical, deposited on O‐CNT, low‐temperature heat treatment	2018	[50]
Bridging S_2_^2−^, vacant coordination sites at Mo centers	**Mo_3_**	Heterogeneous, photochemical, deposited on TiO_2_	2022	[51]
Bridging S_2_^2−^	a‐MoS_x_, Mo‐precursor: (NH_4_)_2_[MoS_4_]	Heterogeneous, electrochemical, deposited on Mo or GC electrodes	2016	[52]
Bridging S^2−^and Mo═O intermediates	Amorphous MoS_3_, Mo‐precursor: (NH_4_)_2_[MoS_4_]	Heterogeneous, electrochemical, deposited on CP	2020	[53]
Bridging S_2_^2−^	**Mo_2_**	Heterogeneous, electrochemical, deposited on FTO or GC electrodes	2015	[54]
Mo‐centered				
Mo^IV^‐centered mechanism (Mo‐hydride)	a‐MoS_x_, Mo‐precursor: (NH_4_)_2_[MoS_4_]	Heterogeneous, electrochemical, deposited on FTO electrodes	2016	[46]
Trapped Mo^III^‐H	Precatalytic a‐MoS_x_, Mo‐precursor: (NH_4_)_2_[MoS_4_]	Heterogeneous, electrochemical, deposited on a planar Au^[^ ^55^ ^]^ or GC^[^ ^56^ ^]^ electrode	2022	[55, 56]
Others				
S_2_^2−^‐centered mechanism	[Mo^IV^S_2_(2,6‐*bis*(1,1‐*bis*(2‐pyridyl(ethyl)pyridine)]^2+^	Heterogeneous, electrochemical, deposited on GC or Hg drop electrodes	2012	[58]
Protonated S_2_^2−^, Mo═O (as proton relay)	[Mo^VI^O(S_2_)_2_L_2_]^−^/[Mo^VI^O(S_2_)_2_L] (L = pic^[^ ^59^ ^]^ pym,^[^ ^59^ ^]^ bpyR (R = H,^[^ ^60^, ^61^ ^]^ *^t^* Bu,^[^ ^60^ ^]^ OMe^[^ ^60^ ^]^))	Homogeneous, electrochemical^[^ ^59^ ^]^	2016, 2017	[59, 60, 61]
Mo^IV^─H mechanism and neighboring bridging hydrosulfide intermediates	[Mo_3_(µ_3_‐S)(µ_2_‐S_2_)_3_(S_2_CNR_2_)_3_]I (R = Me, Et, *^t^* Bu, CH_2_C_6_H_5_)	Homogeneous, photochemical	2019	[44]
S‐centered mechanism	[Mo_2_O_2_S*_y_*]^2−^; [(H)Mo_2_O_2_S*_x_*]^−^ (*y* = 6, 5; *x* = 6, 5, 4)	Homogeneous, gas phase CID and FT‐ICR MS	2022	[62]
S‐centered mechanism, PCET	[M_2_O_2_(µ‐S)_2_(S_2_)(S*_x_*)_2_]^2−^ (M = Mo, W; *x* = 2, 4)	Heterogeneous, electrochemical, deposited on GC electrodes	2019	[32]

Notes:

PCET: proton‐coupled electron transfer; pic: picolinate; pym: pyrimidine‐2‐carboxylate; bpy: 2,2′‐bipyridine; CID: collision‐induced dissociation; FT‐ICR MS: Fourier transform ion cyclotron resonance mass spectrometry; IRMPD: infrared multiple photon dissociation; O‐CNT: oxidized multiwalled carbon nanotubes; GC: glassy carbon; CP: carbon paper; FTO: fluorine‐doped tin oxide.

#### 2.2.1 Sulfide/Disulfide Mechanisms

Experimental and theoretical evidence highlighting the importance of the disulfide ligands were reported by Fantauzzi, Jacob, Streb and colleagues, who studied the light‐driven HER using **Mo_3_** as a catalyst.^[^ ^40^ ^]^ The authors showed that under catalytic conditions in the presence of water, partial or complete exchange of terminal disulfide ligands with water ligands leads to several **Mo_3_** derivatives (which can be described as [Mo_3_S_13−_ *_x_* (H_2_O)*_x_*]^(2−^ *^x^* ^)−^) that coexist in the reaction solution (Figure 2b). Experimental and theoretical analyses indicated that the number of water ligands in such a cluster controls the HER activity: while the fully exchanged species [Mo_3_S_7_(H_2_O)_6_]^4+^ showed only marginal HER activity, Mo_3_‐core surrounded by a mixture of terminal disulfide and water ligands showed the highest reactivity. The introduction of terminal halide ligands also resulted in species with low HER activity. The study further used DFT calculations to explore the energetics of hydrogen evolution on the different water‐substituted **Mo_3_** species. The authors concluded that the energetics of the Volmer step for hydrogen adsorption at bridging disulfide ligands and for forming Mo‐hydride species are similar, suggesting that both mechanistic paths are energetically feasible. These results could be interpreted as a first theoretical indication that **Mo_3_** species are capable of undergoing sulfide/disulfide as well as molybdenum hydride mechanisms in HER catalysis Section 3.

**Figure 3:**
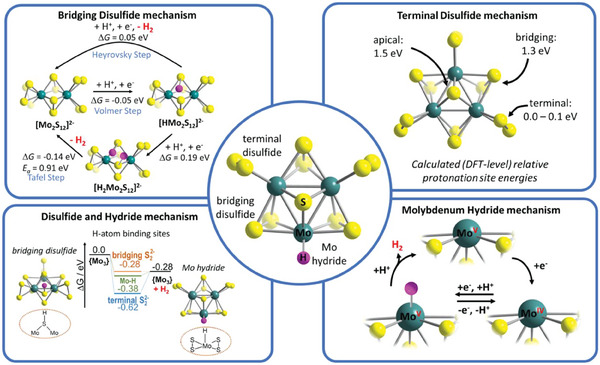
Illustration of possible active sites in the thiomolybdate prototype Mo_3_, and proposed mechanisms at these active sites.

Supporting evidence for a terminal disulfide ligand exchange mechanism was reported by Ončák and co‐workers.^[^ ^47^ ^]^ The groups combined mass spectrometric collision‐induced dissociation (CID) experiments with theoretical DFT calculations to examine possible paths to **Mo_3_** HER activity. The authors observed that even under low energy CID conditions, a range of sulfur/sulfide fragments could be generated from **Mo_3_**. The authors also note that the **Mo_3_**‐based species created by CID show high reactivity with water, as observed by the formation of oxothiomolybdates as secondary species. As the mass spectrometric analyses did not allow unambiguous assignment of the type of sulfide ligands removed, the authors performed a subsequent study employing **Mo_3_** derivatives where all terminal disulfides were replaced by halides (chloride, bromide, and iodide). Gas‐phase fragmentation studies showed that loss of the terminal halides is the energetically most feasible fragmentation route, lending further support to the concept of preferred exchange at the terminal ligand sites.^[^ ^48^ ^]^ In a follow‐up work, Ončák and colleagues further linked thermochemical data calculated by DFT to experimental Fourier‐transform ion‐cyclotron resonance mass spectrometry (FT‐ICR MS) and infrared multiple photon dissociation (IRMPD) spectroscopy data measured for a singly protonated [HMo_3_S_13_]^−^ species.^[^ ^49^ ^]^ The authors reported that under their conditions (in the gas phase), only the **Mo_3_**‐based species with a singly protonated terminal disulfide ligand is observed. Calculations show that this species is thermochemically significantly more stable (by more than 1 eV) compared with **Mo_3_** isomers where protonation occurs on the bridging disulfide or the apical sulfide position (**Figure** **3**b). This study, therefore, suggests that protonated terminal disulfides are key HER intermediates. However, the authors also point out that these gas‐phase studies cannot be directly compared with solution analyses, as many effects in the condensed phase can impact the stability and formation pathways of the intermediate and catalytic species.

In contrast to this series of studies focusing on terminal disulfide‐based HER reactivity for thiomolybdate‐derived catalysts, Joh and colleagues proposed that bridging sulfur ligands play a key role as HER active sites in heterogeneous systems accessed by thermal treatment of **Mo_3_**.^[^ ^50^ ^]^ The authors used a combination of thermogravimetric analysis (TGA) and X‐ray photoelectron spectroscopy (XPS) to link weight loss under heating to changes of the deconvoluted sulfur XPS signal to differentiate between apical/bridging and terminal sulfur positions. The authors propose that upon heating, first the apical sulfide ligand is removed, followed by the loss of the bridging ligands. Catalytic analyses of the resulting materials (supported on carbon nanotubes [CNTs]) showed that higher thermal treatment leads to lower electrocatalytic HER activity. However, further studies are required to fully appreciate the impact of sulfur removal versus structural/morphological changes caused by the heat treatment. Careful electrochemical studies, e.g., using electrochemical impedance spectroscopy or electrochemically active surface area determinations, could help to shed light on this intriguing result.

Very recently, Cherevan and colleagues reported a one‐step deposition of **Mo_3_** on TiO_2_ particles for light‐driven HER. The authors assigned the stable attachment to the formation of Mo─O─Ti bonds accompanied by the loss of most of the terminal disulfide ligands of **Mo_3_**.^[^ ^51^ ^]^ Using XPS and thermal analyses together with pre‐ and postcatalytic materials comparison, the authors concluded that molybdenum centers with vacant coordination sites, Mo‐oxo species or bridging disulfides are likely HER‐active sites. In addition, the authors note that under catalytic conditions, polymerization of individual **Mo_3_** species on the TiO_2_ surface is observed, highlighting that operando studies are required to follow the temporal development of these catalysts as the reaction progresses.

Yeo and co‐workers used operando Raman spectroelectrochemistry to assess possible active sites in a‐MoS_2_ films synthesized using **Mo_3_** as precursor.^[^ ^52^ ^]^ The group observed a characteristic Raman signal assigned to the S─H stretching of a Mo─S─H moiety. The assignment was supported by H/D isotope labeling and DFT calculations. Furthermore, the authors did not observe any Mo‐hydride vibrations, which could be expected for a molybdenum hydride HER mechanism. Following a similar approach, Park and colleagues emphasize the importance of Mo═O intermediates in HER catalysis for a‐MoS_x_ derived from thiomolybdate chains.^[^ ^53^ ^]^ The heterogeneous material was obtained by simple polymerization of monomeric thiomolybdate [MoS_4_]^2−^ species. Resonance Raman spectroscopy and extended X‐ray absorption fine structure (EXAFS) analyses showed that under catalytic conditions, formation of Mo(O)*_x_* species (*x* = 1, 2) is observed. The authors suggest that both, the presence of these Mo(O)*_x_* species and the presence of Mo^V^ units are important for binding and stabilizing protons on the reactive Mo─S site. They propose that the molybdenum‐oxo groups could be responsible for proton management, e.g., by stabilizing hydrogen‐bound intermediates via hydrogen bonding and by facilitating hydrogen atom transfer to the reactive disulfide ligands.

Chen and co‐workers used the small **Mo_2_** thiomolybdate as a model HER electrocatalyst by drop‐casting **Mo_2_** on electrode surfaces.^[^ ^54^ ^]^ The authors used DFT calculations to study the high HER activity observed for **Mo_2_**. Their theoretical analyses show a calculated Gibbs free energy for hydrogen atom adsorption to the bridging disulfide ligands (−0.05 eV), which is close to the thermoneutral optimum of 0 eV. Experimental electrochemistry (Tafel slope analysis) and theoretical calculations both support that H_2_ formation proceeds via a Volmer–Heyrovsky mechanism, while high activation barriers are calculated for the alternative Volmer–Tafel mechanism (Figure 3a; Scheme 1).

#### 2.2.2 Mo‐Hydride Mechanisms

In contrast to these sulfur‐based mechanisms, Tran et al. proposed a Mo‐based HER mechanism, based on electrochemistry, resonance Raman spectroscopy, and electron paramagnetic resonance (EPR) spectroscopy as well as DFT calculations.^[^ ^46^ ^]^ The authors studied samples of **Mo_3_** as nanoparticles or a‐MoS_x_ films deposited on electrodes. Based on their results, they propose that under reductive electrochemical conditions, a pre‐catalytic electrochemical activation occurs where Mo^IV^ centers with a vacant coordination site are generated on **Mo_3_**. These Mo^IV^ centers are reported to represent the catalytically active site (Figure 3d). Theoretical studies support the possibility of Mo^IV^‐hydride moieties as active sites of **Mo_3_**‐derived amorphous MoS_x_ films. In addition, the authors reported that during HER electrocatalysis, resonance Raman data show that the signal intensity for bridging disulfide ligands is reduced, and the signal for terminal disulfides is completely lost. Also, the apical sulfide ligand signal is shifted, and the formation of Mo‐oxide species is observed. Thus, the authors suggest that the resulting amorphous materials are best described by the formula MoS_2+_ *_x_*.

Very recently, Bau et al. reported the presence of Mo^III^ hydride species in **Mo_3_**‐derived a‐MoS_x_ deposited on electrode surfaces.^[^ ^55^ ^]^ The authors report the observation of isotropic EPR spectra, which can be interpreted as a Mo^III^‐H species formed by electrochemical reduction starting from Mo^IV^‐based amorphous molybdenum sulfide. In‐depth (in situ/operando) analyses, e.g., using X‐ray absorption spectroscopy (XAS) methods are expected to provide further evidence for this striking initial hypothesis. The authors built on this work and proposed that Mo^III^ hydrides play an important role in many molybdenum‐based HER electrocatalysts. Their hypothesis is based on experimental and theoretical analysis of a range of catalytic systems. The authors propose that (partial) oxidation of the Mo centers is a key deactivation mechanism, since Mo^III^‐oxo species are difficult to reduce to the active Mo^III^‐centers, and oxo ligands also limit proton diffusion to the Mo site.^[^ ^56^ ^]^

#### 2.2.3 Organo‐Functionalized Thiomolybdate Derivatives

Research has also focused on systems beyond pure molybdenum sulfides. In particular, modification of thiomolybdates with organic ligands has been used to control reactivity. In pioneering work, Appell et al. reported the electrocatalytic homogeneous HER of binuclear [(CpMoS)_2_S_2_CH_2_] and related species at nearly 100% current efficiency and low overpotentials (Figure 1, middle).^[^ ^57^ ^]^ In mechanistic studies, the author team proposed that the rate‐determining step appears to be the elimination of dihydrogen which can occur via neighboring hydrosulfide ligands or mixed hydrosulfido/Mo‐hydride species.

Chang and co‐workers explored the electrocatalytic homogeneous HER activity of the complex [(PY_5_Me_2_)Mo^IV^S_2_]^2+^ (PY_5_Me_2_ = 2,6‐*bis*(1,1‐*bis*(2‐pyridyl)ethyl)pyridine), which is a molecular model for the terminal Mo‐disulfide moiety (Figure 1, middle).^[^ ^58^ ^]^ The authors observed that the complex can be reduced by at least two electrons. In the presence of protons under reductive electrochemical conditions, a strong catalytic wave, and H_2_ evolution at nearly 100% Faradaic efficiency is observed. Voltammetric analyses show that the first reduction of the complex is proton‐coupled, allowing the authors to propose that this process corresponds either to the formation of a protonated, one‐electron‐reduced Mo‐disulfide species, or the breaking of a Mo─S bond between the molybdenum center and the disulfide ligand. Notably, reference experiments with the species [(PY_5_Me_2_)MoO]^2+^ where the disulfide ligand is replaced with a terminal oxo group show much lower HER activity, highlighting the importance of the disulfide ligand, either as an active site or as a modulator to facilitate reduction of the complex.

A different, elegant model system has been reported by Wu and co‐workers.^[^ ^59^, ^60^, ^61^ ^]^ The group developed a mononuclear species, [Mo^IV^O(S_2_)_2_ *L*] (*L* = organic ligand, e.g., picolinate,^[^ ^59^ ^]^ pyrimidine‐2‐carboxylate or 2,2′‐bipyridine‐derivatives^[^ ^60^, ^61^ ^]^), as an active‐site model for a‐MoS_x_. For the 2,2‐bipyridine‐derivative, the authors propose an electrocatalytic Volmer‐Heyrovsky type HER mechanism, which proceeds by two‐electron reduction and protonation of a disulfide (to yield a sulfide, S^2−^ and a hydrosulfide, HS^−^).^[^ ^61^ ^]^ The hydrosulfide ligand acts as a “hydride” mimic and reacts with a second proton in a Mo‐oxo‐mediated Heyrovsky step, resulting in H_2_ evolution and regeneration of the original catalyst. In a subsequent study, the group demonstrated the importance of the organic 2,2‐bipyridine (bpy) ligand. Modification of the electronic structure of the ligand by attaching electron donating groups had a direct impact on the nucleophilicity of the disulfide ligands on the Mo center. This, in turn, modulated the protonation affinity of the disulfides, which affected the HER performance. The authors also demonstrate that the disulfide ligands are the redox‐active sites in this system, while the molybdenum center retains its oxidation state (+IV) even under highly reductive conditions. This could be explained by the presence of the electronegative oxo ligand, which prevents further Mo reduction.

Schmehl and colleagues studied a **Mo_3_** derivative, in which the three terminal disulfide ligands were selectively replaced with organic dithiocarbamates (Figure 2c).^[^ ^44^ ^]^ This model compound is well suited to explore light‐driven homogeneous HER activity in the absence of terminal disulfides. The authors used mass spectrometry to follow changes in the catalyst composition under turnover conditions and observed that the actual catalytic species is formed by conversion of the bridging disulfides to sulfide ligands. Catalyst “poisoning” experiments using strongly coordinating solvents such as DMF showed that at least one vacant coordination site at a Mo center is required for HER to proceed. Based on this observation and DFT calculations, the authors propose a species featuring a Mo^IV^ hydride and adjacent bridging hydrosulfide as the most likely intermediate formed before H_2_ release.

#### 2.2.4 Mixed Oxothiomolybdates

The introduction of oxo‐ligands in thiomolybdates has a major impact on the reactivity of the resulting oxothiomolybdates (vide supra),^[^ ^47^, ^51^, ^53^ ^]^ which led to seminal studies exploring their HER‐function. Following the suggestion that protonated terminal disulfides are important HER intermediates for **Mo_3_**, Ončák and colleagues used theoretical calculations to study potential H atom binding sites to [Mo_2_O_2_S*_x_*]*^n^* ^−^ in different charge states together with H_2_ elimination pathways. The authors identified several H atom adsorption sites as well as energetically favored H_2_ elimination pathways. This flexibility, combined with the ease of electron attachment and removal, appears to be the crucial property that makes molybdenum oxysulfides such good HER catalysts.^[^ ^62^ ^]^ Miras and co‐workers reported the electrocatalytic HER activity of the dinuclear compounds [Mo_2_O_2_(S)_2_(S_2_)(S*_x_*)]^2−^ (*x* = 2, 4; Figure 1) when deposited on GC electrodes.^[^ ^32^ ^]^ Computational analysis of the systems suggested a Volmer–Heyrovsky mechanism where proton‐coupled two‐electron‐reduction leads to a reductive cleavage of a disulfide ligand and the formation of two hydrosulfides. The authors note that the exact mechanism of the Heyrovsky step in these models is still not fully understood, so a complete rationalization of the H_2_ release requires further studies. In addition, DFT calculations suggest that the oxo ligand plays an important role in stabilizing the reduced catalyst species by accepting negative charge density due to the high electronegativity of the oxygen atom. In addition, Nadjo and co‐workers explored the electrocatalytic homogeneous HER activity of mixed oxothiomolybdate rings, such as [Mo_8_S_8_O_8_(OH)_8_(oxalate)]^2−^.^[^ ^63^ ^]^ The group reported electrochemical studies where catalytic hydrogen evolution was observed under reducing conditions in DMF solution in the presence of strong as well as weak acids.

As shown in Table 1, when summarizing the findings from Section 3.2, there is a general trend that under homogeneous conditions, most reports favor terminal disulfide active sites, while under heterogenized conditions, bridging disulfides are mainly discussed as active sites. Mo‐hydride species are most often described when amorphous a‐MoS_x_ is studied, while for thiomolybdates featuring organic ligands, a variety of possible active sites have been reported. While each individual study provides a huge contribution to improve the general understanding of underlying mechanistic processes, stability and identification of catalytically active sites, many of the studies seem contradictory, and there is no general consensus in the field in terms of the true reaction mechanism (or mechanisms). This is due to the fact that the molybdenum sulfide HER mechanism is highly dependent on the exact reaction conditions, the type of support used, and on the exact structure of the cluster. Hence, further work including the design of suitable model systems, as well as combined experimental and theoretical studies—ideally involving in situ and operando investigations—are required to fully understand the complex mechanisms involved in hydrogen evolution by thiomolybdates.

### 2.3 Heterogenization of Thiometalates

In this section, we will pick up on initial discussions from Sections 3.1 and 3.2 concerning the prospects and benefits of thiometalate heterogenization. In this context, heterogenization describes either the anchoring of individual thiometalate clusters on the surface of solid‐state supports, the incorporation of thiometalates within (micro)porous matrices or the deposition of clusters followed by their chemical conversion into nanostructured amorphous or crystalline particles. Similar to the case of other molecular inorganics such as polyoxometalates,^[^ ^64^ ^]^ surface‐anchoring of thiometalate clusters can rely on electrostatic (due to their ionic charge), covalent (due to exchange of the sulfide/disulfide ligands), or weaker noncovalent interactions between cluster and the support surface. From the synthetic point of view, heterogenization can be achieved using a range of immobilization techniques including dip‐coating, solvothermal deposition, layer‐by‐layer assembly, and electrodeposition.^[^ ^64^ ^]^ Note that depending on the deposition methods and conditions used, the conversion of molecular thiomolybdates into more complex nanostructures—often a‐MoS_x_—is possible and should be critically assessed when studying and reporting thiomolybdate‐based electrocatalysts.

Heterogenization of thiomolybdates is a fast‐moving research direction due to several reasons: deposition of thiomolybdates on electrically conductive substrates or semiconductors facilitates their use in (photo)electrochemical HER as heterogeneous materials, which is an advantage for scaling and technological processing. Furthermore, heterogenization could lead to a stabilization of the thiomolybdate clusters, e.g., by preventing fast ligand exchange or providing structural stabilization during the catalytic redox cycles. In addition, molecule–support interactions could be used to further fine‐tune reactivity and stability of thiomolybdates, e.g., by utilizing the supports for managing proton and/or electron transfer. Finally, the use of semiconductors as supports opens new avenues to combine heterogenization and photosensitization in one material, making the traditional use of noble metal‐based photosensitizers (e.g., [Ru(bpy)_3_]^2+^) obsolete. These and other aspects of thiomolybdate heterogenization will be next discussed from the application point of view in Section 4.

## 3 Applications of Heterogenized Thiomolybdate Clusters

This section aims to scrutinize the applications of surface‐anchored thiomolybdate clusters. The analysis focuses on the catalytic performance as well as on details of the surface‐anchoring, the stability of the resulting system, and the fate of the cluster, from deposition through to catalysis. In particular, we highlight the emerging use of in situ/operando analyses as well as the combination of experiment and theory to rationalize the observed reactivity trends. In Section 3.1, we focus on electrocatalysis, while Section 3.2 describes selected examples of photocatalytic deployment. In Section 3.3, recent examples where heterogenized thiomolybdates were used for reactions and processes other than HER are summarized.

### 3.1 Electrocatalysis

Solid‐state molybdenum sulfides are well known for their excellent electrocatalytic HER performance and are discussed as replacements for platinum‐based HER catalysts. In particular, MoS_2+_ *_x_* systems with an S‐to‐Mo ratio higher than two have been reported to be excellent electrocatalysts for the HER.^[^ ^65^ ^]^ This section explores how thiomolybdate‐based heterogeneous materials are designed, tuned and deployed as high‐performance HER electrocatalysts.

Along with TONs that are indicative of the catalyst stability, turnover frequencies (TOFs)—a measure of the rate of catalysis per active site—are key figure of merits for the HER performance and will be used throughout this section as an indicator for the intrinsic HER activity measured for the thiomolybdate clusters. Note that for most of the heterogenized catalysts discussed here, the calculation of TOFs and TONs assumes that every cluster present in the catalyst is available for the reaction. Thus, the use of TON and TOF—particularly in heterogenized systems, requires careful thought and critical analysis of the system studied.^[^ ^66^, ^67^, ^68^ ^]^ Also note that all potentials mentioned throughout this chapter (and those in **Table** **2**) are reported versus the reversible hydrogen electrode (RHE) and all overpotentials are reported at the current density of 10 mA cm^−2^ unless stated otherwise. Nevertheless, we strongly advise the reader to always be aware of the substrate used for the electrode, as different supporting materials can have varied electrochemical surface areas for the same geometric surface area, thus affecting the reported performance indicators. The challenges of benchmarking electrocatalytic reactions have been comprehensively reviewed by Jaramillo and co‐workers.^[^ ^69^ ^]^

**Table 2:** Supported thiomolybdates in electrocatalytic HER: summary table showing prominent examples of thiomolybdate clusters implementation, along with the supports used, deposition applied, and HER performance indicators including Tafel slopes, overpotentials, TOF values as well as major experimental conditions

Cluster	Support	Deposition technique	Experimental conditions	Tafel slope [mV dec^−1^]	Overpotential at 10 mA cm^−2^ vs RHE [mV]	TOF at 200 mV [s^−1^]	Year	Ref.
MoS_2_								
MoS_2_ NPs	Au(111)	PVD of Mo in H_2_S background	H_2_SO_4_, pH 0.2	55	–	0.02	2007	[10]
MoS_2_	rGO | GCE	Drop casting	0.5 m H_2_SO_4_	41	–	–	2011	[85]
Monodispersed MoS_2_	Au^[^ ^110^ ^]^	Incubation	0.5 m H_2_SO_4_	69	180–190	–	2013	[86]
		Drop casting		100	–		2013	[86]
Mo_x_S_4_								
[Mo_3_S_4_]^4+^	C‐xc72	Drop casting	0.5 m H_2_SO_4_, pH 0.4	120	200	0.07	2008	[38]
CuMoS_4_	GCE	Drop casting	0.1 m H_3_PO_4_, pH 7	–	–	–	2012	[87]
Mo_2_/Mo_3_/W_2_								
Mo_3_	GP/HOPG	Drop casting	0.5 m H_2_SO_4_	40/57	180	1	2014	[71]
Mo_3_	Au | GC	Spray coating	0.1 m HClO_4_	58	–	0.47	2017	[72]
Mo_3_	Ag | GC			48	–	0.27	2017	[72]
Mo_3_	GC			45	–	0.15	2017	[72]
Mo_3_	Cu | GC			66	–	0.045	2017	[72]
Mo_3_	rGO‐CNTs	Solvothermal	0.5 m H_2_SO_4_	67.4	179	–	2017	[76]
Mo_3_	HOPG	Electrodeposition	0.5 m H_2_SO_4_	37	200	–	2017	[78]
Mo_3_	O‐CNTs	Physical mixture	0.5 m H_2_SO_4_	40	137	2.5 at 250 mV	2018	[50]
Mo_2_	FTO	Drop casting	0.5 m H_2_SO_4_	39	161	1.6	2015	[54]
[Mo_2_O_2_S_6_]^2−^	GCE	Drop casting	1 m H_2_SO_4_	52	114	0.12	2019	[32]
[Mo_2_O_2_S_8_]^2−^			1 m H_2_SO_4_	55	116	0.12	2019	[32]
[W_2_O_2_S_8_]^2−^			1 m H_2_SO_4_	100	227	0.13	2019	[32]
MoS_3_								
a‐MoS_3_‐Mo_1_‐derived	Carbon paper	Drop casting	0.5 m H_2_SO_4_	46	167	0.01	2020	[53]
a‐MoS_3_‐Mo_2_‐derived			0.5 m H_2_SO_4_	47	170	0.004	2020	[53]
a‐MoS_3_‐Mo_3_‐derived			0.5 m H_2_SO_4_	52	196	0.0006	2020	[53]
MoS_3_	FTO	Spray casting	1 m H_2_SO_4_	45	190	–	2011	[88]
MoS_3_	FTO	Drop casting	1 m H_2_SO_4_, pH 0	61	200	–	2011	[88]
MoS_3_	GCE	Drop casting	1 m H_2_SO_4_	50	200	–	2011	[88]
Others/composites								
bachera‐MoS_2_/MWCNT	Ag	Drop casting + in situ reduction of MoS_3_	1 m H_2_SO_4_	40	–	–	2013	[89]
Ni‐P/MoS_x_	FTO	Electrodeposition	1 m KOH	64	140	–	2017	[90]
MoS_x_	FTO	Electrodeposition	1 m KOH	140	260	–	2017	[90]
MoS_x_/MWCNT	GCE	Drop casting	0.5 m H_2_SO_4_	62	–	–	2016	[91]
MoS_x_/GO	GCE	Drop casting	0.5 m H_2_SO_4_	60	180	0.94 at 220 mV	2016	[92]
Pd‐MoS_2_/MWCNT	GCE	Drop casting	0.5 m H_2_SO_4_	54	120	0.05 at 30 mV	2017	[93]
MoS_2_/CN_x_	GCE	Drop casting	0.5 m H_2_SO_4_	52	158	–	2017	[94]

Notes:

NPs: nanoparticles; PVD: physical vapor deposition; rGO: reduced graphene oxide; GCE: glassy carbon electrode; C‐xc72: vulcan carbon; GP: graphite paper; HOPG: highly oriented pyrolytic graphite; O‐CNTs: oxidized carbon nanotubes; FTO: fluorine‐doped tin oxide; MWCNT: multiwalled carbon nanotubes.

Further, throughout this section we will use Tafel slope analysis as a tool to gain insights into the rate‐limiting steps in electrocatalytic HER. For example, a Tafel slope below 30 mV dec^−1^ is indicative of the Tafel reaction (H* recombination) to be the rate‐limiting step. Similarly, a Tafel slope around 40 or 120 mV dec^−1^ suggests Heyrovsky (electrochemical H_2_ desorption) or Volmer (discharge) step be the rate‐limiting step, respectively (Scheme 1).^[^ ^70^ ^]^

#### 3.1.1 Electrocatalytic HER Activity of Heterogenized Thiomolybdates

This section summarizes the initial reports on molecular mimics of MoS_2_ electroactive toward HER, along with factors affecting their performance. Chorkendorff and co‐workers were the first to test cubane‐type [Mo_3_S_4_]^4+^ clusters supported on carbon materials (Vulcan xc72 and highly oriented pyrolytic graphite [HOPG]) for the electrocatalytic HER.^[^ ^38^ ^]^ For [Mo_3_S_4_]^4+^/Vulcan system, the authors reported a low HER onset potential of ≈150 mV, similar to that of nanoparticulate MoS_2_. However, the authors observed drops in current density upon multiple scans without any change in onset potential, which they attribute to possible dissolution of the clusters. Further investigation using scanning tunneling microscopic (STM) imaging was conducted for the drop‐cast [Mo_3_S_4_]^4+^/HOPG system revealing agglomeration mounds—possibly corresponding to cluster deposits or products of its degradation—which the authors related to the weak interactions between the clusters and the substrate surface. This assumption was further strengthened based on postcatalytic XPS analysis of [Mo_3_S_4_]^4+^/HOPG, which revealed almost no peaks corresponding to the Mo 3d of the pristine cluster suggesting unstable nature of its attachment. To circumvent this issue, HOPG surface was activated by electrochemical oxidation to introduce ─OH and ─COOH groups able to act as anchoring sites for [Mo_3_S_4_]^4+^ cations. Combining this strategy with STM imaging to reveal the surface density of the clusters, the authors reached a TOF of 0.07 s^−1^ per [Mo_3_S_4_]^4+^ molecule, as compared to 0.02 s^−1^ (per edge site determined via STM imaging) measured for the reference MoS_2_ sample.

**Figure 4:**
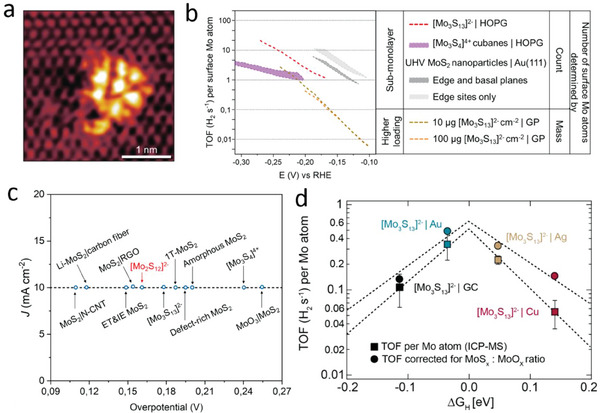
Supported thiometalates in electrocatalysis. a) Scanning tunneling microscopy (STM) image of a single Mo_3_ cluster on HOPG support. b) TOF plot comparing electrochemical HER performance of Mo_3_/HOPG and Mo_3_/GP with other MoS_x_ catalysts. c) Overpotential comparison between various Mo─S based catalysts. d) HER volcano plot showing TOF_MoOx_ corrected using ICP‐MS. (a,b) Reproduced and adapted with permission.^[^ ^71^ ^]^ Copyright 2014, Nature Chemistry. (c) Adapted with permission.^[^ ^54^ ^]^ Copyright 2015, Wiley‐VCH GmbH. (d) Reproduced with permission.^[^ ^72^ ^]^ Copyright 2017, American Chemical Society.

Almost half a decade later, Besenbacher and co‐workers reported excellent electrocatalytic performance by **Mo_3_** which was drop‐cast onto an anodized HOPG surface (**Figure** **4**a). XPS analysis confirmed stable attachment of the clusters onto the surface without any transformation.^[^ ^71^ ^]^ **Mo_3_** dispersed over HOPG exhibited a low overpotential of ≈180 mV and a Tafel slope of ≈57 mV dec^−1^, indicating H* recombination to be the rate‐determining step (Scheme 1). However, to obtain a better comparison to reported literature, the authors loaded the clusters onto graphite paper (GP) increasing the loading from 10 to 100 µg cm^−2^. They observed all Tafel slopes to be around 38–40 mV dec^−1^, which indicates the Heyrovsky (desorption) step to be rate limiting. To further elucidate the reason for the change in HER mechanism and avoid any effects due to the change in support, the authors measured the Tafel slopes of different loadings of clusters onto a single support, glassy carbon (GC). They observed a Tafel slope of 40 mV dec^−1^ at higher loadings whereas a Tafel slope of ≈60 mV dec^−1^ was observed for very low loadings similar to the case of HOPG. Therefore, the authors suggested this change in the mechanism is independent of the support and rather could be due to closer packing of the cluster (and thus active sites) which affects the H‐adsorption and chemical rearrangement of the intermediate steps during HER. As to the intrinsic HER activity of the clusters, the authors reported a high TOF of 3 s^−1^ per Mo atom (i.e., 1 s^−1^ per **Mo_3_**) at −200 mV for the **Mo_3_**/HOPG architecture (Figure 4b). However, they did observe a fivefold lower TOF when depositing higher loadings of the clusters onto GP, which they attributed to shielding of the catalytic sites due to cluster agglomeration. As an important contrast to the [Mo_3_S_4_]^4+^ work conducted by Chorkendorff and co‐workers,^[^ ^38^ ^]^ here the authors observed no loss in catalytic performance, which suggests a more stable binding of **Mo_3_** to anodized HOPG compared with [Mo_3_S_4_]^4+^. As a consequence, upon accelerated HER testing (1000 cyclic voltammetry (CV) cycles between −0.3 and +0.2 V), the **Mo_3_**/HOPG composite showed only a slight decrease in current density and a small increase in total impedance.

Building on these results, Wu and co‐workers explored the electrochemical performance of the dimeric **Mo_2_** deposited onto FTO substrate via layer‐by‐layer assembly (involving positively charged polyquaternium‐6 interlayer) and drop‐casting, aiming for sub‐monolayers and higher catalyst loading, respectively.^[^ ^54^ ^]^ They reported an even higher TOF of 3.2 s^−1^ per Mo atom (which could be translated to 1.6 s^−1^ per **Mo_2_** cluster based on total **Mo_2_** loading) at −200 mV, while the overpotential required to reach a current density of 10 mA cm^−2^ for **Mo_2_** was found to be only 161 mV, outcompeting both previously mentioned [Mo_3_S_4_]^4+^ and **Mo_3_** that showed an overpotential of 240 and 180 mV, respectively, when deposited on HOPG (Figure 4c). To explain this difference in performance, the authors theoretically modeled H adsorption on the unsupported clusters in question. They calculated the change in free energy for **Mo_2_** and **Mo_3_** as −0.05 and −0.08 eV, respectively: these values are significantly closer to the theoretical optimum (0.0 eV) compared with data reported earlier for [Mo_3_S_4_]^4+^ (0.4 eV).^[^ ^54^ ^]^ Therefore, based on these initial works, dimeric **Mo_2_** seems to outperform the previously reported MoS_x_‐based HER electrocatalysts due to favourable adsorption‐desorption thermodynamics. However, more studies and direct comparison between **Mo_2_** and **Mo_3_** under identical experimental conditions is necessary to identify their active HER sites and unravel structural features which control their HER performance.

#### 3.1.2 Catalyst–Support Interactions

The choice of the substrate is essential for providing robust anchoring and improved electronic coupling between catalyst and substrate. A bulk of earlier work on supported MoS_2_‐based catalytic phases for HDS, HDO and HDM catalysis already demonstrated a synergistic effect between the two components and importance of the support choice.^[^ ^73^, ^74^ ^]^ In this section, we discuss the current understanding of how the choice of substrates affects the stability and electrocatalytic performance of heterogenized thiomolybdates.

Building on their earlier studies on **Mo_3_**, Jaramillo and co‐workers carried out a follow up study correlating the HER activity of **Mo_3_** with the type of support material used.^[^ ^72^ ^]^ The group studied electrocatalytic HER performance of a thin layer of cluster deposited on four substrates, i.e., pristine glassy carbon (GC), Cu on GC (Cu/GC), Ag on GC (Ag/GC), and Au on GC (Au/GC). The authors deliberately kept the catalyst loading low to be able to observe the effects of the catalyst–support interactions as these tend to have a short range.^[^ ^75^ ^]^ By keeping the catalyst loading low the authors also avoided any detrimental effects related to cluster aggregation. The results of XPS analyses of the supported catalysts were virtually identical to the pure **Mo_3_** precursors, suggesting that the heterogenization did not majorly affect the structure of the deposited **Mo_3_**. Tafel slope analyses for all samples were in the range of 40 to 60 mV dec^−1^, so that no clear insights into the difference in underlying rate‐determining steps were possible. In contrast, a marked impact of the substrate on the TOF was observed, and the resulting TOF values at −200 mV were measured to be 0.47 s^−1^ (Au/GC), 0.27 s^−1^ (Ag/GC), 0.15 s^−1^ (GC), and 0.045 s^−1^ (Cu/GC), respectively (Figure 4d). The authors carried out DFT calculations considering long range van der Waals forces to account for the effect of the strength of the cluster adhesion on the support on its activity.^[^ ^75^ ^]^ They correlate the observed TOFs to changes in Gibbs free energy for hydrogen adsorption on the cluster (for each support) and arrived at the conclusion that the relationship between the two follows a volcano‐plot trend well known for other HER electrocatalysts.^[^ ^2^ ^]^

**Figure 5:**
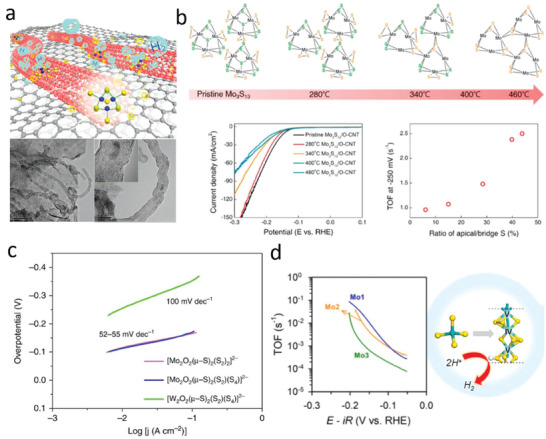
Supported thiometalates in electrocatalysis. a) Schematic representation of the Mo_3_/rGO‐CNTs composite structure with TEM images of Mo_3_/rGO‐CNT taken after 1000 HER cycles. b) Proposed structural evolution and electrochemical HER performance with TOFs of thermally annealed Mo_3_/CNT composites at 280, 340, 400, and 460 °C. c) Tafel plots of different Mo‐ and W‐based oxothiometalate clusters. d) TOFs of Mo_1_‐, Mo_2_‐, and Mo_3_‐derived MoS_3_ (its model on the right). (a) Reproduced with permission.^[^ ^76^ ^]^ Copyright 2017, American Chemical Society. (b) Reproduced with permission.^[^ ^50^ ^]^ Copyright 2018, American Chemical Society. (c) Reproduced with permission.^[^ ^32^ ^]^ Copyright 2019, Nature Communications. (d) Reproduced with permission.^[^ ^53^ ^]^ Copyright 2020, American Chemical Society.

Following similar concepts, Gao and co‐workers reported an increase in electrocatalytic HER performance at low catalyst loadings when immobilizing **Mo_3_** onto highly conductive reduced graphene oxide (rGO)–CNT aerogels (**Figure** **5**a),^[^ ^76^ ^]^ reporting an HER overpotential of 179 mV. Attachment of the clusters to the aerogels was demonstrated using multiple techniques including EDS mapping and Raman spectroscopy. The authors reported a low Tafel slope of 60–70 mV dec^−1^, similar to the case reported by Jaramillo and co‐workers in the best‐performing **Mo_3_** on Au/GC and Cu/GC.^[^ ^72^ ^]^ The authors employed zeta potential measurements and XPS analyses to gain insights into the immobilization mechanism. Zeta potential measurements revealed a negatively charged rGO‐CNT surface, which—considering the anionic **Mo_3_** clusters in solution—renders electrostatic attachment unlikely. XPS analysis showed a characteristic S─O binding peak allowing the authors to suggest strong cluster attachment via covalent linkage to the O‐containing functional groups on the nanocarbon surface. However, we note that molybdenum sulfides and thiomolybdates can feature oxidized sulfur species (e.g., sulfate) left from the synthesis or formed during catalysis.^[^ ^77^ ^]^ Thus, care must be taken when interpreting the origin of any S─O signals, e.g., in XPS. Thus, further studies might be required to fully appreciate the type of attachment present in the **Mo_3_**/rGO‐CNT composites. For example, an alternative attachment scenario could take place via the **Mo_3_** ligand exchange mechanism discussed in Section 3, which shows that charge‐neutral or even cationic cluster species could be accessed. As to the electrocatalytic performance of the **Mo_3_**/rGO‐CNT composite, although the authors did not report the TOF values, the small Tafel slope, low charge transfer, and dispersion resistances indicate a fast reaction rate corresponding to efficient HER performance and low overpotentials. Moreover, the authors report a negligible change in activities even after 1000 CV cycles, further confirming the robust nature of the **Mo_3_**/rGO‐CNT electrocatalyst (Figure 5a).

Li and co‐workers took a different approach to strengthen the linkage between the cluster and substrate.^[^ ^78^ ^]^ Contrary to using drop‐casting or impregnation methods, they electrodeposited **Mo_3_** onto HOGP in the form of flakes of varying thicknesses by changing the deposition times. Note that under mild electrodeposition conditions, anionic **Mo_3_** clusters can transform into an amorphous MoS‐like structure that is largely made up of the original {Mo_3_S_7_} subunits.^[^ ^79^ ^]^ In line with this, XPS analysis complemented by atomic emission spectroscopy and XAS measurements, confirmed the structural integrity of the cluster after deposition. At optimized film thicknesses (beyond 1500 s when through‐film resistance starts dominating the process), the as‐derived **Mo_3_**/HOPG attained an onset potential of 130 mV with a Tafel slope of 37 mV dec^−1^ and showed no signs of Mo leaching even after 3000 CV cycles, thereby confirming the durability of the supported electrocatalyst. Compared to simple drop‐cast films, electrodeposited **Mo_3_**/HOPG electrodes showed a much lower charge transfer resistance demonstrating remarkable electron transport.

In addition, Assaud and co‐workers reported an interesting study demonstrating the effect of thiomolybdate cluster interactions with carbon‐based supports commonly used in PEM water electrolysis.^[^ ^80^ ^]^ They observed a strong decrease in onset potential from −439 to −124 mV when moving from graphene‐ to Vulcan‐supported [Mo_3_S_4_]^4+^‐based clusters. Furthermore, the authors also reported a much lower overpotential required for [Mo_3_S_4_]^4+^/Vulcan as compared to [Mo_3_S_4_]^4+^/graphene to reach a current density of 30 mA cm^−2^. Since these overpotentials already consider surface roughness characteristics of the two supports and the current densities were already normalized per electrochemically active surface area, the authors proposed that this difference in performance is due to stronger physisorption of [Mo_3_S_4_]^4+^ on the Vulcan surface.

#### 3.1.3 Role of Cluster Structure

Finally, this section outlines the studies performed to gain an in‐depth understanding of how the composition, structure, and origin of the thiomolybdate species can be linked to their HER performance.

Traditionally, **Mo_3_** clusters have been synthesized from (NH_4_)_6_[Mo_7_O_24_] precursor via a method reported by Müller and co‐workers.^[^ ^27^, ^28^, ^81^ ^]^ However, there are several other synthetic methods of **Mo_3_** formation using molybdenum trisulfide (MoS_3_) as proposed by Müller and Weber.^[^ ^82^, ^83^ ^]^ Building on this, Joh and co‐workers investigated electrochemical HER activity of MoS_3_‐derived **Mo_3_** by supporting it onto oxidized multiwalled carbon nanotubes.^[^ ^50^ ^]^ The authors observe an overpotential of 137 mV and a Tafel slope of 40 mV dec^−1^. To further understand the role of each component they next explored the possibility of **Mo_3_** cluster polymerization by studying the effect of thermally assisted conversion of **Mo_3_**. The authors demonstrated that heat‐treatment of **Mo_3_** at different temperatures up to 460 °C results in materials which show variable TOFs in the electrocatalytic HER reaction (Figure 5b). Based on these catalytic data and complemented by DFT studies, the authors proposed bridging disulfide ligands to be the likely active HER site, as was detailed in Section 3.2.1. We note that the authors propose that the heat treatment at specific temperatures can be used to trigger selective structural changes (e.g., loss of the apical sulfide ligands). However, this level of assignment requires further detailed analysis, e.g., by vibrational spectroscopy or mass spectrometry to verify this hypothesis. Alternative processes might include (partial) cluster degradation and conversion into amorphous molybdenum sulfides.

In another prominent work, Miras and co‐workers investigated the impact of the structural and stoichiometric ratio of the chalcogen to transition metal on HER performance by examining a broader set of oxothiometalate clusters.^[^ ^32^ ^]^ Focusing specifically on the thiomolybdates, the authors compared [Mo_2_O_2_(S)_2_(S_2_)_2_]^2–^ and [Mo_2_O_2_(S)_2_(S_2_)(S_4_)]^2−^ (Figure 1, right) drop‐cast onto GC electrodes. Both clusters exhibited similar Tafel slopes in the range of 52–54 mV dec^−1^ and a low overpotential of about 114 mV, which demonstrates a negligible effect of the terminal sulfide groups in the cluster on the HER mechanism and performance (Figure 5c). On contrary to this conclusion, the authors estimated a TOF of 0.12 s^−1^ for both oxothiometalates. This value is significantly lower as compared to TOFs reported for other similar clusters (Table 2), thus still leaving an open question to the stability and potential transformation of oxothiometalates under turnover conditions.

Joo and co‐workers explored the effect of Mo oxidation state in the MoS_x_ family of clusters on the overall HER.^[^ ^53^ ^]^ They prepared amorphous MoS_3_ polymeric chains using [MoS_4_]^2−^, **Mo_2_**, and **Mo_3_** as monoatomic, diatomic, and triatomic Mo precursors, respectively. The electrodes were prepared by drop casting the precursors onto carbon paper, followed by thermal treatment at 120 °C to initiate polymerization. The samples were labeled as Mo_1_‐, Mo_2_‐, and Mo_3_‐derived MoS_3_, depending on the precursor chemistry. After the synthesis, Mo_1_‐derived films showed the lowest overpotential of 167 mV, closely followed by 170 mV for Mo_2_‐derived and the highest overpotential of 196 mV for Mo_3_‐derived electrocatalysts. However, Tafel slopes for each of them was within the range of 46–52 mV dec^−1^. Further characterization revealed that Mo_1_‐derived MoS_3_ had a higher electrochemical active surface area as compared to other electrocatalysts, thus justifying its superior HER performance amongst the three (Figure 5d). Since both **Mo_2_** and **Mo_3_** clusters have been used as precatalysts (precursors) in this work, no further conclusion about the impact of thiomolybdate molecular structure on HER activity could be drawn from the results. However, this work demonstrates that thiomolybdates can be used as precursors which allow to obtain a variety of structurally different MoS_x_ electrocatalysts.

Overall, Table 2 highlights, that the HER performance of thiomolybdates depends strongly on the type of the cluster and type of the support used with overpotential values reported in the range between 120 and 200 mV (at 10 mA cm^−2^ current density). This range is generally higher when compared to molybdenum carbides or phosphides; however, MoS_2_‐based nanostructures are known for higher intrinsic activity (i.e., TOF values)^[^ ^84^ ^]^ still making thiomolybdates and their derivatives attractive.

### 3.2 Photocatalysis

**Figure 6:**
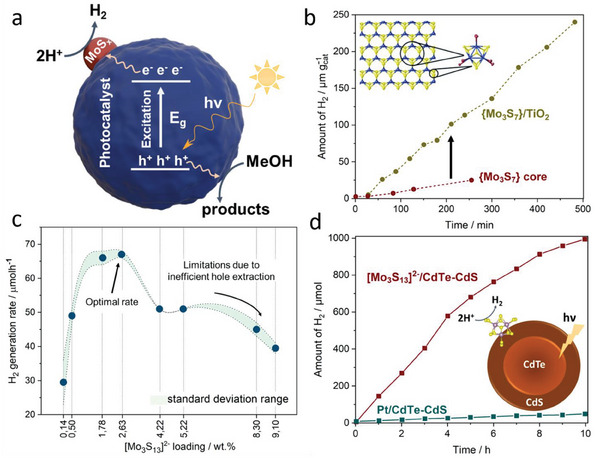
Supported thiometalates in photocatalysis. a) Illustration of charge transfer from photocatalyst to the thiometalate co‐catalyst upon light illumination. E_g_: energy bandgap; MoS_x_: any thiometalate cluster. b) Hydrogen generation profiles of heterogenized {Mo_3_S_7_}/TiO_2_ (5 wt% cluster loading, 1 g L^−1^) and {Mo_3_S_7_}‐core containing complex (homogeneous, 0.31 g L^−1^) in acetone/H_2_O mixture obtained in the presence of 0.1 m Na_2_S and 0.02 m Na_2_SO_3_. c) Hydrogen evolution rate plotted against the Mo_3_ cluster loading on TiO_2_: volcano type profile is highlighted. d) Amount of hydrogen produced by Mo_3_/CdTe@CdS (Mo_3_ concentration: 4.6 × 10^−6^ m) and Pt/CdTe‐CdS under optimal reaction conditions of pH = 2.5 from 20 mg mL^−1^ ascorbic acid aqueous solution (20 mL). (b) Adapted with permission.^[^ ^95^ ^]^ Copyright 2015, Wiley‐VCH. (c) Reproduced with permission.^[^ ^51^ ^]^ Copyright 2022, American Chemical Society. (d) Adapted with permission.^[^ ^96^ ^]^ Copyright 2016, American Chemical Society.

Soon after the first studies on thiomolybdate HER electrocatalysis, the light‐driven catalysis community started exploring these cluster species as molecular co‐catalysts. Starting with the most common UV‐active TiO_2_ as a photoactive support, a range of narrow‐band semiconductors and (micro)porous hosts have been employed to design efficient and robust heterogeneous photoactive composites (**Figure** **6**a). Section 4.2.1 discusses these studies with a focus on photocatalytic HER by thiomolybdate anchored on TiO_2_ semiconductors, while Section 4.2.2 focuses on visible‐light‐active semiconductor supports. In Section 4.2.3, the photoreactivity of thiomolybdates heterogenized in porous matrices is discussed.

#### 3.2.1 Thiomolybdate Clusters as HER Co‐Catalysts

Several prominent works have been reported over the past years, in which heterogenized thiomolybdates have been used for light‐driven HER. In 2014, Llusar and co‐workers reported the use of [Mo_3_S_7_]^4+^ fragments which were heterogenized onto TiO_2_ to act as a co‐catalyst for photocatalytic HER.^[^ ^95^ ^]^ To facilitate covalent anchoring of the cluster on the oxide surface, the authors employed the {Mo_3_S_7_}‐core‐containing complex [Mo_3_S_7_Br_4_bpy(CO_2_Me)_2_], (bpy(CO_2_Me)_2_ = 4,4′‐dimethyldicarboxylate‐2,2′‐bipyridine, see Figure 1, right). Based on electrochemical and spectroscopic measurements, the authors suggest that the molecular species serves as a precatalyst and is reductively converted to a more active catalyst species when in contact with sulfite/sulfide species in the reaction solution. The nature of these reduced species was unraveled by XPS analysis, according to which the final electroactive material is composed of Mo^IV^ atoms coordinated to S^2−^ moieties, suggesting a MoS_2_‐based compound. The resulting MoS_2_/TiO_2_ composite showed promising visible light‐driven HER with no performance loss even after 8 h of operation (Figure 6b).

More recently, Cherevan and colleagues investigated the attachment modes and loading‐dependent photocatalytic HER performance of the non‐modified **Mo_3_** cluster which was anchored onto TiO_2_.^[^ ^51^ ^]^ The authors proposed that **Mo_3_** deposition follows a monolayer adsorption and involves the formation of Mo─O─Ti coordinative bonds while maintaining the overall molecular structure of **Mo_3_**. The authors further explored the photocatalytic HER performance of **Mo_3_**/TiO_2_ composites with different cluster loadings revealing optimal activity at around 3 wt% of **Mo_3_** (Figure 6c). As revealed by radical‐trapping photoluminescence (PL) spectroscopy, the drop for higher loading values is related to the availability of the support‐solution interface required for the efficient hole scavenging. The authors also performed long‐term HER studies which showed stable photocatalytic H_2_ evolution rates. These results are in strong contrast to the work discussed previously^[^ ^95^ ^]^ and suggest that native thiomolybdates without any organo‐functionalization are able to undergo direct binding with oxide‐based supports and further act as structurally stable co‐catalysts for HER.

In related studies, Du and colleagues explored the surface attachment, optical properties, photocatalytic HER performance and stability of **Mo_2_** as a co‐catalyst when deposited onto anatase‐type TiO_2_.^[^ ^97^ ^]^ XPS analyses indicated that **Mo_2_** attaches covalently to the TiO_2_ support by replacement of the terminal S_2_^2−^ ligands. Optimum HER activity was achieved at 3 wt% loading. The authors additionally reported PL data for **Mo_2_**/TiO_2_ composites, which documented improvement in charge separation (low PL intensity and higher excited state lifetime when compared to pure TiO_2_), while complementary photoelectrochemical measurements showed higher photocurrent for the optimum **Mo_2_**/TiO_2_ composite, which further confirms improved charge separation dynamics and suggests efficient charge extraction by the surface‐bound **Mo_2_** species.

#### 3.2.2 Extension to Visible‐Light‐Active Supports

For light‐driven HER, it is crucial to investigate support materials that can maximize the absorption of solar light.^[^ ^98^ ^]^ One of the most promising classes to this end are semiconducting CdX (X = S, Se, Te) quantum dots (QDs), as their light absorption properties can be fine‐tuned by modulating their dimensions and composition. Zhao and co‐workers employed CdTe/CdS core/shell QDs as support for **Mo_3_** and verified the cluster anchoring by a combination of high‐resolution transmission electron microscopy (HRTEM) and XPS analyses.^[^ ^96^ ^]^ The authors proposed that the adsorption of **Mo_3_** occurs via the Cd‐terminated faces as well as surface defects, including Cd adatoms and S vacancies of QDs. The HER activity of **Mo_3_**/CdTe‐CdS composite was almost constant over more than 10 h of illumination. The authors also report the HER performance to be significantly higher compared with a benchmark Pt co‐catalyst loaded on CdTe‐CdS QDs (Figure 6d). However, the team also emphasized that the **Mo_3_**/CdTe‐CdS composites were not stable below the optimal pH of 2.5 triggered by the precipitation of QDs and the weakened interaction between the two components. Also, the study reports that increasing **Mo_3_** loading above a certain level results in the drop of HER activity, most likely due to light blocking by the catalyst.

**Figure 7:**
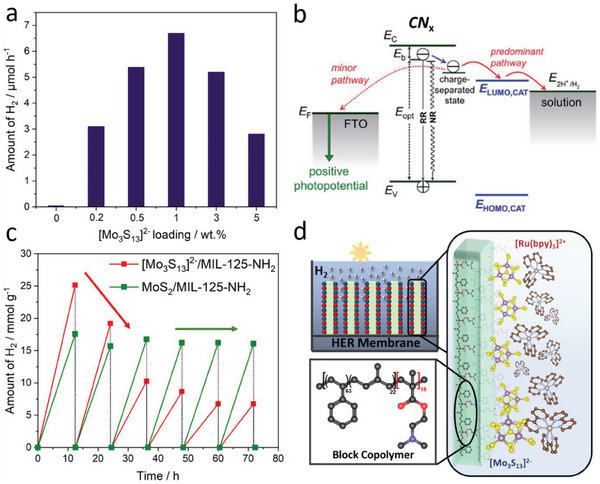
Supported thiometalates in photocatalysis. a) Effect of different loadings of Mo_3_ in Mo_3_/CN_x_ composite on the amount of hydrogen produced. b) Scheme showing the change in photopotential at FTO/CN_x_ photoelectrode surface in the presence of Mo_3_ catalyst. E_c_: conduction band edge; and E_v_: valence band edge; RR: radiative recombination from emissive (band‐edge) states; NR: nonradiative recombination from nonemissive (shallow‐trap) excitonic states; E_opt_: optical bandgap of CN_x_; E_b_: exciton binding energy; CAT: Mo_3_ catalyst. c) Comparison of Mo_3_/MIL‐125‐NH_2_ and MoS_2_/MIL‐125‐NH_2_ in terms of their stability under HER experimental conditions. d) Design of photocatalytic hydrogen evolution system based on nanoporous block copolymer membrane that is electrostatically co‐anchoring Mo_3_ catalyst and [Ru(bpy)_3_]^2+^ photosensitizer. (a) Adapted with permission.^[^ ^100^ ^]^ Copyright 2017, Royal Society of Chemistry. (b) Reproduced with permission.^[^ ^101^ ^]^ Copyright 2020, Royal Society of Chemistry. (c) Adapted with permission.^[^ ^102^ ^]^ Copyright 2018, American Chemical Society. (d) Adapted with permission.^[^ ^77^ ^]^ Copyright 2020, Royal Society of Chemistry.

Carbon nitride (CN_x_) is another promising narrow band gap semiconductor often used by the community as an efficient metal‐free heterogeneous light absorber. Driven by the idea to utilize the anionic charge of **Mo_3_** for the attachment, Wang and co‐workers used melon‐derived mesoporous CN_x_—that was rendered positively charged via facile protonation^[^ ^99^ ^]^—as a support for the attachment.^[^ ^100^ ^]^ The **Mo_3_**/CN_x_ composite was characterized using TEM and XPS measurements, which confirmed the monodispersed nature of the clusters. Similar to the works discussed above, the authors found **Mo_3_** to act as efficient HER co‐catalyst (**Figure** **7**a): (a) when supported on insulating SiO_2_, **Mo_3_** was unable to generate any H_2_ and (b) when neat (uncharged) CN_x_ was used as a support, much lower HER activities were recorded. These data correlate well with the conclusions drawn in Section 4.1 and strongly suggest the importance of strong (electrostatic) interaction between catalyst and support, which is critical to achieve optimal HER performance.

While many photosystems have demonstrated that **Mo_3_** can act as co‐catalysts for light‐driven HER, Streb and colleagues provided a unique perspective on the electrostatically assembled **Mo_3_**/CN_x_—using amorphous carbon nitride support—aiming to shed light on the charge transfer dynamics between the two components as well as unravel the reaction kinetics under turnover conditions.^[^ ^101^ ^]^ The authors utilized steady‐state and time‐resolved PL spectroscopy to reveal that increasing cluster loading in **Mo_3_**/CN_x_ composites leads to enhanced charge separation at the interface. As such, for smaller cluster loadings, **Mo_3_** seems to selectively bind to the amine groups. As these are associated with shallow trap states, this binding tends to repopulate the emissive excitonic states, thereby increasing the PL intensity and lifetime. Higher cluster loadings, on the other hand, increase the nonradiative decay, which dissociates the shallow trapped excitons into charge carriers and leads to improved charge separation, causing the PL emission intensity and lifetime to decrease. These findings provide a valuable perspective to explain the loading‐dependent HER performance observed in this and other works. The second relevant observation was delivered by the transient photopotential measurements able to elaborate the effect of **Mo_3_** co‐catalyst on the charge separation extent. As shown in Figure 7b, the photopotential is negative for the FTO‐supported CN_x_, which the authors attributed to its insulating behavior and the poor electrical connection between CN_x_ and FTO. Conversely, a positive photopotential was recorded when **Mo_3_** was attached to the CN_x_ surface, suggesting the efficient extraction of photoexcited electrons by the cluster. These data suggest that surface‐anchored **Mo_3_** strongly affects the exciton dissociation and mediates the transfer of trapped electrons from CN_x_ to the reactants. However, as the addition of **Mo_3_** had no impact on the ultrafast (sub‐ns) photoinduced kinetics in the CN_x_, the authors also note that the electron extraction to **Mo_3_** occurs on a relatively longer (ns‐s) time scale.

#### 3.2.3 Thiomolybdates within Porous Hosts

Metal–organic frameworks (MOFs) have recently attracted the attention of the community as organic–inorganic hybrid materials comprised of metal ions or metal‐oxo clusters linked by bridging organic ligands to form multidimensional solid‐state networks. Their modular assembly and tunable pore structure and pore size makes them ideally suited for heterogenization of thiomolybdate HER catalysts.^[^ ^103^ ^]^ This principle has been explored by Stylianou and colleagues who used the visible‐light‐active MIL‐125‐NH_2_ MOF as a support to compare (co‐)catalytic HER performance of **Mo_3_** and 1T‐MoS_2_ nanoparticles (Figure 7c).^[^ ^102^ ^]^ Their data suggest **Mo_3_**/MOF to be more active compared to MoS_2_/MOF when similar co‐catalyst loadings were used. The authors attributed this observation to the improved interaction of molecular clusters with the MOF surface along with their inherently higher catalytic activity discussed previously in Section 4.1. However, the authors also found **Mo_3_**/MOF to be less stable in long‐term photocatalytic tests compared to 1T‐MoS_2_/MOF composites, which could be attributed to structural instability of the **Mo_3_** under alkaline pH conditions.^[^ ^100^, ^101^ ^]^ More investigations are required to elaborate on this important issue.

In another prominent example, Streb and co‐workers were the first to use soft‐matter‐based nanoporous block copolymer membranes with cationic surface groups as heterogeneous supports for electrostatic anchoring of **Mo_3_** catalyst and [Ru(bpy)_3_]^2+^ photosensitizer for light‐driven HER (Figure 7d).^[^ ^77^ ^]^ The functionalized polymer membranes were analyzed using scanning electron microscopy (SEM), energy dispersive X‐ray spectroscopy (EDX), and XPS analyses, which suggest that one of the terminal S_2_^2−^ ligands is replaced with a water ligand after attachment to the membrane, in line with the behavior observed previously.^[^ ^40^ ^]^ The copolymer‐supported **Mo_3_**/[Ru(bpy)_3_]^2+^ photosystem generated H_2_ for at least 20 h of illumination. However, the authors also observed that a considerable amount of H_2_ was stored in the pores of the polymer membrane and could be recovered after the catalytic process. The authors further reported that **Mo_3_** did not leach from the membrane; however, they noted significant loss of the photosensitizer after 10 h of irradiation. Their data suggest that even at low Ru‐photosensitizer loadings, the membrane is able to preserve its HER activity, possibly by mediating the electron transfer between photosensitizer and catalyst within the membrane.

### 3.3 Other Applications

Apart from electrocatalytic and photocatalytic applications involving thiometalates, most recent works have been exploring the heterogenized clusters in thermal catalysis, and selective ion capture. As such, Zhang and co‐workers have immobilized **Mo_2_** and **Mo_3_** on mesoporous SiO_2_ (SBA‐15) and applied the composites for thiophene hydrodesulfurization (HDS), a process used to remove sulfur‐containing compounds from petroleum.^[^ ^104^ ^]^ The results showed that both thiomolybdates can be successfully deposited on SBA‐15, while maintaining their structure. The stability of the supported catalysts was assessed through TGA, which revealed that **Mo_2_**/**Mo_3_** are likely to retain their sulfur ligands during catalysis. The composites were also tested for hydrogen adsorption using temperature‐programmed reduction, which indicated the hydrogenation of terminal and bridging sulfur species to be the likely intermediate step of the HDS process. Overall, the study demonstrates the successful loading and effective catalytic performance of the thiomolybdates in HDS. The findings further provide insights into the role of sulfur species in the catalytic process and contribute to the development of supported thiometalate clusters for other catalytic applications.

In another outstanding study, Ma, Kanatzidis and colleagues used **Mo_3_** to prepare composites for the selective extraction of silver from copper‐rich minerals.^[^ ^105^ ^]^ The authors incorporated **Mo_3_** into the matrix of a conjugated polypyrrole (ppy) backbone by ion‐exchange reaction and demonstrated exceptional selectivity and high efficiency of the resulting **Mo_3_**/ppy in capturing Ag^+^ and Hg^2+^ ions at concentration levels <1 ppb. The mechanism for silver removal involves two synergistic pathways: direct binding and in situ reduction. In the former, the **Mo_3_** clusters release S^2−^ ions, which bind with Ag ions to form Ag_2_S complexes, effectively removing Ag from the solution. In the latter, the Mo^IV^ ions in Mo_3_ act as reducing agents, converting Ag^+^ ions to metallic silver while being oxidized to Mo^VI^. This reduction reaction occurs alongside the binding of Ag^+^ ions to S^2−^ ions, resulting in the deposition of silver crystals on the **Mo_3_**/ppy composite surface. The results suggest that composites based on **Mo_3_**—and, potentially, other thiometalates—can offer a promising approach for selective silver extraction and removal of toxic metal ions from complex aqueous environments.

Parallel to the implementation of thiometalates as reduction co‐catalysts for HER, several works have employed composites containing such clusters to promote various oxidation reactions. In an early set of studies, **Mo_3_** was employed as co‐catalyst for photocatalytic dye degradation using visible‐light‐active BiOBr and Bi_2_WO_6_ substrates prepared through a hydrothermal method. Both nanocomposites demonstrated excellent photocatalytic activity—comparable to state‐of‐the‐art Pt co‐catalysts—which was primarily attributed to the ability of **Mo_3_** to efficiently promote the extraction of photoexcited electrons thus indirectly affecting the lifetimes of electron‐hole pairs and hole utilization efficiency.^[^ ^106^, ^107^ ^]^ In addition to this, the authors highlighted that, compared to commonly used Pt, **Mo_3_** shows enhanced sulfur tolerance and can thus be used effectively in processes involving sulfur species, such as in desulfurization or sulfur‐containing wastewater remediation.

In a more recent example of thiomolybdate application, Xufang and co‐workers fabricated a novel **Mo_3_**/TiO_2_ photocatalyst for the oxidation of acetone to CO_2_.^[^ ^108^ ^]^ The CO_2_ production was investigated with **Mo_3_**/TiO_2_ composites containing different loadings of **Mo_3_**: 0.6, 1.1, 1.7, and 3.4 wt%. The authors found that among these composites, the highest amount of CO_2_ was produced with 1.7**Mo_3_**/TiO_2_, which was ascribed to the optimal loading of clusters over titania surface promoting better photoinduced charge separation. Importantly for benchmarking, the 1.7**Mo_3_**/TiO_2_ photocatalyst was found to be more active for acetone mineralization compared to the benchmark Pt/TiO_2_ photocatalyst exhibiting a similar co‐catalyst loading value. In addition, the solution of 1.7**Mo_3_** TiO_2_ composite sprayed onto polypropylene nonwoven fabric produced higher CO_2_ amounts compared to the pristine TiO_2_/fabric, which one more time highlights the effect of **Mo_3_** in the structure. These polypropylene‐coated nonwoven fabrics coated with **Mo_3_**/TiO_2_ composite have been used as an efficient photocatalyst for indoor air purification.

## 4 Conclusions and Outlook

This review placed special emphasis on recent developments in thiomolybdate catalysis, particularly with respect to combined experimental and theoretical studies that shed light on reaction mechanisms, active sites and potential degradation pathways, as well as on new strategies for thiometalate heterogenization on functional substrates and their emerging applications. Two comprehensive summary Tables were provided to highlight the current state‐of‐the‐art: Table 1 examines active sites of thiomolybdate clusters, Table 2 documents that heterogenized thiomolybdates are highly active, structurally versatile HER promoters and that their molecular nature can be used as a tool to tune their activity.

Despite a number of proof‐of‐concept demonstrations and promising results that are already been recorded, the authors of this perspective have identified a list of critical points that will hopefully help shaping future research directions in the field:

We suggest that more in‐depth mechanistic studies are required to fully understand how thiomolybdates are anchored to heterogeneous supports, and to explore the role of catalyst–support interactions for electrocatalytic and photocatalytic HER. Also, understanding the physical and electronic structure of the catalytically active state of the clusters under turnover conditions is critical for a knowledge‐based development of the field. These themes have been the focus of two recent studies which used time‐resolved methods to gain further insights into thiomolybdate reactivity: Ha‐Thi and colleagues revealed the mechanism of light‐driven catalysis of a {Mo_3_S_4_}‐core containing cluster in solutions,^[^ ^45^ ^]^ while Streb and co‐workers provided important insights into the charge transfer dynamics of surface‐supported **Mo_3_**.^[^ ^101^ ^]^ In another pioneering example, Bozheyev and colleagues used transient surface photovoltage spectroscopy to unravel the electronic properties of WSe_2_‐anchored **Mo_3_** heterostructures.^[^ ^109^ ^]^

Another critical aspect when considering thiomolybdate heterogenization is the stability and fate of the molecules under deposition and reaction conditions. Many studies deliberately use thiomolybdates as molecular precursors for the preparation of MoS_x_ nanostructures or films. However, in other cases, this molecule‐to‐solid‐state compound conversion might occur unintentionally. In both cases, the resulting material needs to be thoroughly characterized, and clear evidence must be provided if the respective manuscript claims that the material used for catalysis is still a molecular species. This can be challenging, particularly when the conversion occurs in situ, under catalytic conditions. Thus, comprehensive pre‐ and postcatalytic analyses—ideally supported by in situ/operando studies—are required to assess the complex processes occurring in this compound class. Key aspects to be focused on are ligand loss/exchange, chemical interaction with the surface, oligo‐ and polymerization of individual clusters and formation of MoS_x_ fragments. As outlined in Sections 2 and 3, there is still much room to explore these questions and provide more insights into improvement when analyzing and reporting these aspects.

In addition to the chemical and structural considerations outlined above, there is an urgent need to provide fundamental insights into the differences in reactivity of different thiomolybdate prototypes, particularly when deposited on identical supports. This would allow the research community to engage in knowledge‐based advanced materials design, based on a full understanding of the underlying reaction and stability mechanisms.

Apart from these critical notes, the authors of this perspective would like to suggest directions of future research:

A current limitation in the field is that most HER research has been focused on the use of a limited number of prototype thiomolybdates, mainly **Mo_2_** and **Mo_3_**. While these studies have provided ground‐breaking new insights and materials, we propose expanding the compound base and exploring the reactivity of advanced cluster types. This includes research on other types of thiometalates, such as thiotungstates,^[^ ^33^ ^]^ mixed‐ligand clusters, e.g., featuring oxo‐ or organic ligands in addition to thio groups,^[^ ^17^ ^]^ as well as selenide‐containing derivatives.^[^ ^110^ ^]^ While these areas are highly promising, they have thus far not received the attention to allow us to draw any final conclusions on the component suitability for HER studies.

State‐of‐the‐art thiomolybdate catalysis research is mainly focused on electrocatalytic or light‐driven HER. However, based on earlier research and on selected recent publications, we suggest that other (thermal) catalytic processes including reductions, oxidations and small molecule activations are intriguing areas to be explored using thiometalates. In a recent example, Llusar and colleagues have combined experimental and theoretical methods to explore the catalytic hydrogenation of azobenzene using cuboidal {Mo_3_S_4_}‐containing clusters.^[^ ^111^ ^]^ The results suggest a mechanism involving homolytic activation of hydrogen at bridging sulfur atoms in the cluster, followed by consecutive hydrogen transfers to azobenzene, resulting in the formation of aniline. The authors also explored the effects of different organic ligands on the catalytic activity of the clusters and provided evidence supporting the sulfur‐centered mechanism, which contributes strongly to a better understanding of the catalytic properties of these and structurally similar thiomolybdates. Along similar lines, mechanistic comparison between HER and hydrodesulfurization catalysis studies can offer a new insight into the function of thiomolybdates. In one example, Suman and co‐workers studied an oxothiomolybdate complex [(Mo_2_O_2_S_4_)(en)(dmf)] and suggested an oxidative addition/reductive elimination mechanism or a ligand‐based insertion reaction—in which sulfur inserts either into the terminal S─S bond or into the Mo─S bond—as possible reaction pathways.^[^ ^112^ ^]^

Finally, the merging of thiomolybdates with functional support materials such as polymers, MOFs, and narrow bandgap semiconductors is also an area which can lead to advanced composites. In one example, Chane‐Ching and colleagues recently immobilized **Mo_2_** derivatives on 2D WS_2_ photoelectrodes to drive photoelectrocatalytic water splitting.^[^ ^113^ ^]^ In MOF‐chemistry, Yaghi and co‐workers were able to build thiomolybdate‐based porous frameworks and demonstrate the HER performance of these pioneering materials.^[^ ^35^ ^]^ We believe that many more functional molecular, hybrid, and solid‐state materials could be realized using thiometalates and their derivatives.

## Conflict of Interest

The authors declare no conflict of interest.
